# Effects of mindfulness-based interventions on sleep quality in university students: a meta-analysis of randomized controlled trials

**DOI:** 10.3389/fpubh.2026.1988584

**Published:** 2026-10-29

**Authors:** Hongwei Wang, Longkang Guo, Junbo Wang, Zhaoxuan Chen, Wei Han

**Affiliations:** 1 College of Competitive Sports, Shandong Sport University, Rizhao, China; 2 Graduate Education College, Shandong Sport University, Jinan, China

**Keywords:** meta-analysis, mindfulness-based interventions, sleep quality, systematic review, university students

## Abstract

**Background:**

Sleep problems are common among university students due to academic stress, lifestyle factors, and psychological distress and may adversely affect physical and mental health, academic performance, and social functioning. Mindfulness-based interventions (MBIs) have increasingly been used as a non-pharmacological approach to improve sleep health; however, previous findings remain inconsistent, and evidence specifically addressing sleep quality in university students remains limited. This study aimed to systematically evaluate the effects of MBIs on sleep quality in university students and explore factors that may account for variations in intervention effects.

**Methods:**

We searched seven databases—PubMed, Web of Science, EBSCO, the Cochrane Library, Scopus, Embase, and ScienceDirect—from inception to June 26, 2026. Randomized controlled trials (RCTs) evaluating MBIs for sleep quality in university students were included. Two reviewers independently conducted study selection, data extraction, and risk-of-bias assessment using the RoB 2 tool. Meta-analysis was conducted in Stata 17.0 using a random-effects model. Subgroup analyses, sensitivity analyses, publication bias assessment, and GRADE assessment were also performed.

**Results:**

Ten RCTs involving 638 university students were included, with 335 participants in the intervention groups and 303 in the control groups. MBIs significantly improved sleep quality [SMD = −0.97, 95% CI (−1.43, −0.51), *p* < 0.001], although substantial heterogeneity was observed (*I*^2^ = 85.1%). Subgroup analyses revealed statistically significant between-group differences with respect to sleep-outcome measurement instrument (*p* = 0.001), intervention type (*p* = 0.009), and session duration (*p* = 0.030), indicating that these variables may partially account for the observed heterogeneity. Sensitivity analyses supported the robustness of the pooled results. Egger’s test revealed no evidence of significant publication bias (*p* = 0.270). The certainty of the evidence was rated as low according to the GRADE framework.

**Conclusion:**

MBIs may improve sleep quality in university students. However, given the substantial heterogeneity and low certainty of evidence, further high-quality RCTs are needed to confirm these findings.

**Systematic review registration:**

CRD420261458068, https://www.crd.york.ac.uk/PROSPERO/view/CRD420261458068.

## 1 Introduction

Sleep is a fundamental physiological need essential for maintaining human health and normal functioning and plays a critical role in cognitive performance, emotional regulation, mental health, and cardiovascular and metabolic function (1). The university years represent a critical transitional period from adolescence to adulthood. During this period, students are exposed to multiple stressors, including academic competition, examination pressure, and career planning, while also commonly engaging in behaviors such as irregular sleep schedules and caffeine consumption, all of which may adversely affect sleep quality. A meta-analysis of 48 studies involving 95,938 undergraduate students reported a pooled prevalence of insomnia symptoms of 46.9% (2). Similarly, a systematic review and meta-analysis of university students in Africa reported pooled prevalences of poor sleep quality of 62.34% among general university students, 64.81% among health science students, and 60.33% among medical students (3), further highlighting the high prevalence of sleep problems among university students. Importantly, poor sleep quality may have a range of adverse consequences. It may impair learning and academic performance among university students (4, 5). In addition, sleep problems are closely associated with anxiety, depression, elevated stress levels, and reduced quality of life (6, 7). Taken together, these findings indicate that sleep problems are common among university students and may adversely affect both their academic performance and their physical and mental health.

Current approaches to improving sleep quality among university students can generally be classified as pharmacological or non-pharmacological interventions. Although pharmacological treatments may alleviate sleep problems to some extent, uncertainty remains regarding their long-term benefits and safety. A study of university students found that continued melatonin use was associated with subsequent increases in sleep disturbance and depressive symptoms (8). Therefore, pharmacological treatment may not be an appropriate routine long-term strategy for managing sleep problems in university students. In contrast, mindfulness-based interventions (MBIs), as a non-pharmacological approach, have increasingly been applied to the management of sleep and psychological health in university populations. These interventions are predominantly meditation-centered and train individuals to observe present-moment bodily sensations, emotions, and cognitive experiences with an open, accepting, and non-judgmental stance, thereby diminishing automatic reactivity to negative thoughts and stressful stimuli (9–11). Greeson et al. reported that the Koru mindfulness program, developed for university students and emerging adults, significantly reduced stress and alleviated sleep problems (12). Another study found that a low-intensity mindfulness intervention improved subjective sleep quality, sleep latency, and habitual sleep efficiency, with some benefits persisting into the later stages of the intervention (13). Collectively, these findings suggest that MBIs may represent a useful adjunctive approach to promoting sleep health in university settings.

Although several randomized controlled trials (RCTs) have suggested that MBIs may improve sleep quality in university students, findings from previous systematic reviews and meta-analyses remain inconsistent. A meta-analysis including five studies found only a small improvement in sleep quality following MBIs, and the pooled effect did not reach statistical significance (14). Another meta-analysis specifically examining mindfulness-based stress reduction (MBSR) among university students included 29 RCTs overall; however, only three studies contributed data on sleep quality, and the pooled effect was likewise not statistically significant (15). In contrast, another meta-analysis including only two studies found that MBIs may significantly improve sleep quality in university students (16). Notably, previous meta-analyses have generally examined MBIs as one subgroup among several psychological interventions or have treated sleep quality as one of multiple secondary outcomes of MBIs. Consequently, systematic evaluations specifically focusing on the effects of MBIs on sleep quality in university students remain relatively limited. Moreover, previous analyses of sleep quality have included relatively few studies and small overall sample sizes, which may have resulted in unstable pooled effect estimates and limited the precision and reliability of their conclusions.

Given these limitations in the existing literature, the present study aimed to systematically evaluate the effects of MBIs on sleep quality in university students and to conduct exploratory subgroup analyses to examine whether intervention characteristics, delivery modalities, and outcome measurement instruments may influence the observed intervention effects. By doing so, this study sought to provide more robust evidence regarding the effectiveness of MBIs and the conditions under which they may be most applicable, while also informing the design of future university-based sleep health interventions and related research.

## 2 Materials and methods

This systematic review and meta-analysis was conducted in strict accordance with the Preferred Reporting Items for Systematic Reviews and Meta-Analyses (PRISMA) 2020 statement and the methodological guidance provided in the Cochrane Handbook (17). The protocol was registered in the International Prospective Register of Systematic Reviews (PROSPERO), with the registration number CRD420261458068.^1^

### 2.1 Literature search strategy

We systematically searched seven databases—PubMed, Web of Science, EBSCO, the Cochrane Library, Scopus, Embase, and ScienceDirect—from inception to June 26, 2026. Searches combined subject headings with free-text terms to identify RCTs evaluating the effects of MBIs on sleep quality in university students. Search terms were developed around four key concepts: “university students,” “mindfulness-based interventions,” “sleep quality,” and “randomized controlled trials.” The detailed PubMed search strategy is presented in Table 1.

**Table 1:** PubMed search strategy.

Steps	Search strategies
#1	(“Sleep Quality”[Mesh]) OR (“Dyssomnias”[Mesh]) OR (“Sleep”[Mesh]) OR (“Sleep Initiation and Maintenance Disorders “[Mesh]) OR (sleeping) OR (daytime dysfunction) OR (difficulty falling asleep) OR (awakenings) OR (Snoring) OR (diurnal tiredness) OR (sleepiness)
#2	(“Mindfulness”[Mesh]) OR (mindfulness-based) OR (mindful) OR (meditation) OR (MBAT) OR (MBSR) OR (MBCT)
#3	(“Students”[Mesh]) OR (student) OR (Undergraduate student) OR (college university student)
#4	(Randomized controlled trial) OR (randomized) OR (placebo) OR (randomized) OR (RCT)
#5	#1 AND #2 AND #3 AND #4

### 2.2 Inclusion and exclusion criteria for literature

The inclusion and exclusion criteria were developed according to the PICOS framework, and studies were systematically screened based on five components: Population, Intervention, Comparison, Outcome, and Study design (18).

#### 2.2.1 Inclusion criteria for literature

1. Population (P): Participants were students enrolled in higher education institutions, including associate degree students, undergraduate students, master’s students, and doctoral students (19).
2. Intervention (I): The intervention group received interventions centered on mindfulness training, including MBSR, mindfulness-based cognitive therapy (MBCT), and mindfulness meditation (MM) (20, 21).
3. Comparison (C): Control conditions included wait-list control, no intervention, usual care, health education, sleep hygiene education, or other comparable control conditions.
4. Outcome (O): Studies were required to report at least one sleep quality-related outcome, assessed using the Pittsburgh Sleep Quality Index (PSQI), Insomnia Severity Index (ISI), Sleep Condition Indicator (SCI), Medical Outcomes Study Sleep Scale (MOS Sleep Scale), or other validated sleep assessment tools.
5. Study design (S): Only RCTs were included because randomization helps minimize confounding and strengthens causal inference regarding intervention effects (22).

#### 2.2.2 Literature exclusion criteria

1. Studies involving participants who were not students enrolled in higher education institutions were excluded.
2. Studies were excluded if mindfulness training was not the core component of the intervention, or if mindfulness was combined with other interventions in a way that did not allow the independent effect of the mindfulness intervention to be evaluated.
3. Studies were excluded if they did not report sleep quality-related outcomes, or if the outcome data were incomplete and could not be extracted or used to calculate an effect size.
4. Studies published in languages other than English were excluded.

### 2.3 Literature screening and data extraction

Study selection and data extraction were independently performed by two reviewers (Junbo Wang and Zhaoxuan Chen). Following the prespecified inclusion and exclusion criteria, the two reviewers independently screened titles and abstracts, assessed potentially eligible articles in full text, and extracted relevant data. Their results were subsequently cross-checked to ensure consistency in study selection and data extraction. Any disagreements between the two reviewers were resolved through discussion with a third reviewer (Longkang Guo) until consensus was reached.

After study selection, the two reviewers independently extracted and verified relevant information from the included studies using a prespecified standardized data extraction form. The extracted information included publication year, country or region, participant characteristics, sample size, mean age, intervention, comparator, mode of intervention delivery, intervention duration, intervention frequency, duration of each intervention session, and sleep quality assessment instruments. For studies with incomplete reporting or unclear data, the original study authors were contacted to obtain additional information. If the required data could not be obtained, they were handled according to the prespecified procedures.

### 2.4 Risk assessment of bias

To assess the methodological quality of the included studies, two reviewers (Junbo Wang and Zhaoxuan Chen) independently evaluated the risk of bias using the Cochrane Risk of Bias 2 (RoB 2) tool. The assessment covered five domains: Randomization process, Deviations from intended interventions, Missing outcome data, Measurement of the outcome, and Selection of the reported result. An overall risk-of-bias judgment was then made based on the assessments across these domains (23). Each domain was rated as “low risk,” “some concerns,” or “high risk.” A study was judged to be at low overall risk of bias when all domains were rated as low risk. A study was judged to have some concerns when one or more domains raised some concerns but none were rated as high risk. A study was judged to be at high overall risk of bias when at least one domain was rated as high risk, or when multiple domains raised some concerns that could substantially reduce confidence in the study findings. After completing the assessments independently, the two reviewers cross-checked their judgments. Any disagreements were reviewed and discussed with a third reviewer (Longkang Guo) until consensus was reached.

### 2.5 Statistical analysis

Data synthesis and meta-analysis were performed using Stata version 17.0 (StataCorp LLC, College Station, TX, United States) (19). Because different measurement instruments were used across the included studies, resulting in differences in measurement units and scoring systems, standardized mean differences (SMD) with 95% confidence intervals (CI) were used as the pooled effect measure (24, 25). Before data synthesis, the direction of all outcome measures was standardized so that lower effect estimates consistently indicated an improvement in sleep quality. Accordingly, an SMD < 0 with a 95% CI entirely below zero indicated that the mindfulness intervention favored improvement in sleep quality among university students. Statistical heterogeneity was assessed using Cochran’s Q test and quantified using the I^2^ statistic and corresponding *p*-value. When *I*^2^ ≤ 50% and *p* ≥ 0.01, heterogeneity among studies was considered not statistically significant, and a fixed-effect model was used for data synthesis. When *I*^2^ > 50% and *p* < 0.01, substantial heterogeneity was considered to be present, and a random-effects model was applied. When evident clinical heterogeneity was identified, subgroup analyses or qualitative synthesis were performed as appropriate (26). Sensitivity analysis was conducted using a leave-one-out approach to assess the robustness of the pooled results. Publication bias was visually assessed using funnel plots and statistically evaluated using Egger’s test, with *p* < 0.05 indicating potential publication bias (27). In addition, the Grading of Recommendations Assessment, Development and Evaluation (GRADE) approach was used to assess the certainty of evidence for the primary outcome (28).

## 3 Results

### 3.1 Results of literature screening

The systematic search of PubMed, Web of Science, Embase, the Cochrane Library, Scopus, EBSCO, and ScienceDirect yielded 1,747 records. An additional two records were identified through manual screening of the reference lists of relevant systematic reviews and meta-analyses, resulting in a total of 1,749 records. After removal of 229 duplicates, the titles and abstracts of the remaining 1,520 records were screened. Of these, 1,481 records were excluded because they were unrelated to the research topic, were not RCTs, or were not published in English. Subsequently, 39 articles were retrieved for full-text assessment and evaluated for eligibility. Of these, 29 were excluded because the study design did not meet the inclusion criteria (*n* = 19), the outcome measures were not eligible (*n* = 9), or the required data were unavailable (*n* = 1). Ultimately, 10 studies met the inclusion criteria and were included in the systematic review and meta-analysis. The complete study selection process is presented in Figure 1.

**Figure 1:**
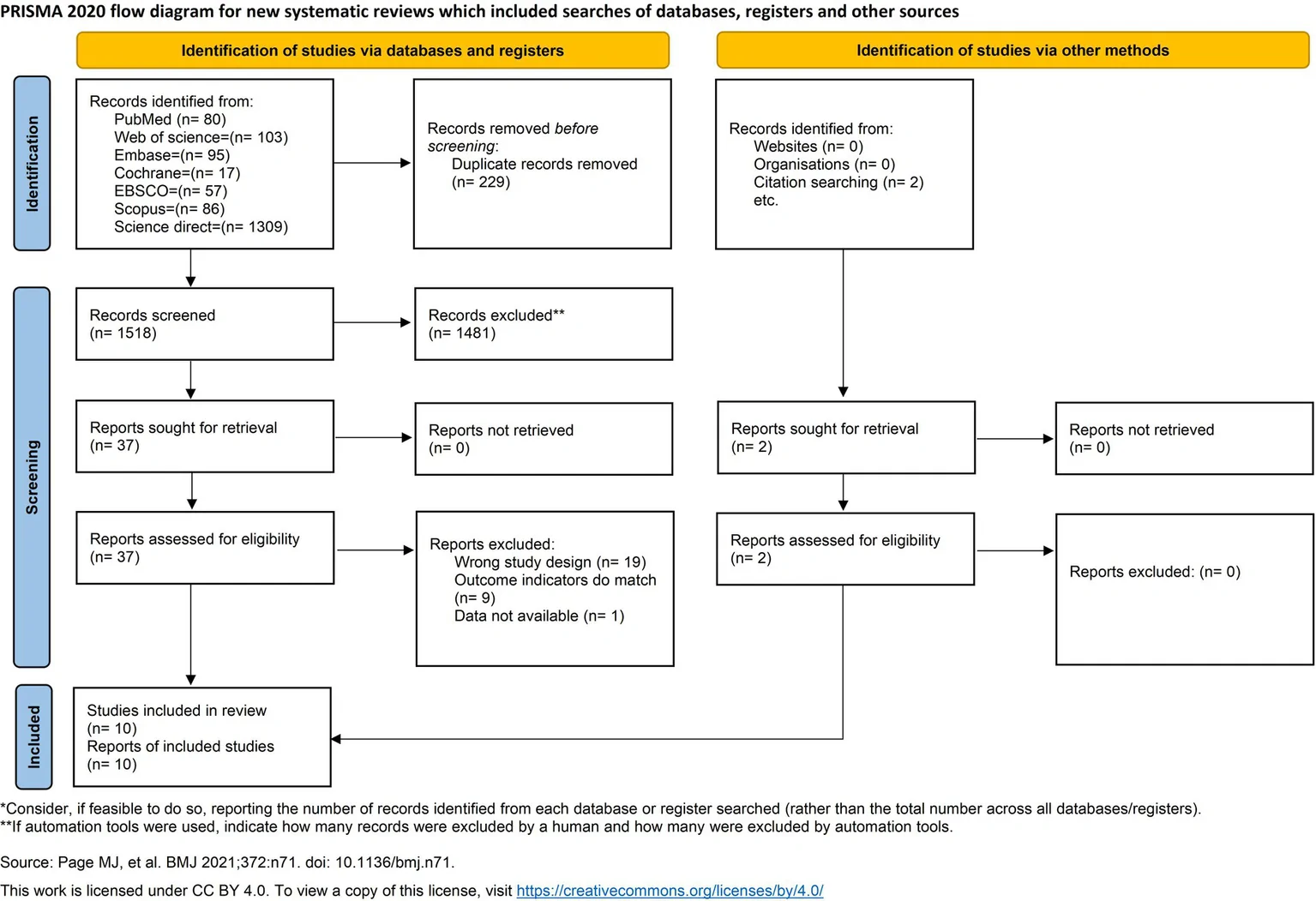
Flow diagram of study selection.

### 3.2 Basic characteristics of the included studies

A total of 10 RCTs published between 2014 and 2026 were included. The studies were conducted primarily in the United States (12, 29–33), China (13, 34, 35), and Canada (36), and involved 638 participants in total, including 335 in the intervention groups and 303 in the control groups. The included interventions were broadly categorized into three types: MBSR, MBCT, and MM. Of the 10 studies, four used MBSR (12, 29, 30, 32), one used MBCT (35), and five used MM (13, 31, 33, 34, 36). Intervention durations ranged from 4 to 8 weeks, intervention frequency ranged from 1 to 7 sessions per week, and individual session durations ranged from 12 to 120 min. Sleep quality was assessed using instruments including the PSQI, MOS SLP9, PROMIS, and SCI. Detailed characteristics of the included studies are presented in Table 2.

**Table 2:** Basic characteristics of the included studies.

Study	Country	Sample size	Age	Duration	Type of intervention	Comparator	Frequency	Population	Session length (min)	Delivery mode	Outcome
Greeson et al. (12)	United States	C: 45 E: 45	C: 24.76 ± 4.15 E: 25.75 ± 6.84	4 weeks	MBSR	Wait-list	1d/week	Undergraduate, graduate, professional students	75	Onsite	MOS SLP9
Dvořáková et al. (29)	United States	C: 53 E:52	18.20 ± 0.40	6 weeks	MBSR	Wait-list	First 2 weeks: 2d Following 4 weeks: 1d	Undergraduate students	80	Onsite	PSQI
Hall et al. (13)	China	C: 12 E: 42	C: 22.64 ± 3.89 E: 22.19 ± 2.08	7 weeks	MM	Wait-list	≥2d/week	Undergraduate and graduate students	25–27	Online	PSQI
Jones et al. (30)	United States	C:12 E:15	18–23	8 weeks	MBSR	No intervention	1d/week	Undergraduate students	75	Onsite	PSQI
Smith et al. (31)	United States	C:7 E:7	Unclear	8 weeks	MM	Wait-list	Daily	Physician assistant students	12	Online	PROMIS
Wu et al. (34)	China	C:30 E:30	C: 19.80 ± 2.41 E: 19.70 ± 2.28	1 month	MM	Wait-list	1d/week	Undergraduate and graduate students	45	Onsite	PSQI
Mirabito and Verhaeghen (32)	United States	C:53 E:58	21.29	4 weeks	MBSR	Wait-list	4d/week	Undergraduate students	75	Online	PSQI
Pickett et al. (33)	United States	C:40 E:37	21.64	4 weeks	MM	Self-monitoring	5d/week	Undergraduate students	15	Online	SCI
Lew et al. (36)	Canada	C:39 E:37	C: 20.64 ± 3.09 E: 21.81 ± 5.76	1 month	MM	Self-monitoring	5d/week	Undergraduate students	20	Online	PSQI
Jiayi et al. (35)	China	C:12 E:12	C: 20.50 ± 1.40 E: 20.30 ± 1.50	8 weeks	MBCT	Sleep hygiene education	1d/week	Undergraduate students	120	Onsite	PSQI

C, Control group; E, Experimental group; MBSR, mindfulness-based stress reduction; MM, mindfulness meditation; MBCT, mindfulness-based cognitive therapy; Unclear, Not reported in the original studies; d/week, Several times a week.

### 3.3 Evaluation of literature quality

The RoB 2 tool was used to assess the risk of bias in the 10 included RCTs. Overall, one study (10.0%) was judged to be at low risk of bias, eight studies (80.0%) were judged to raise some concerns, and one study (10.0%) was judged to be at high risk of bias.

Across individual domains, six studies (60.0%) were rated as low risk and four (40.0%) as having some concerns for the randomization process. For deviations from intended interventions, four studies (40.0%) were rated as low risk and six (60.0%) as having some concerns. For missing outcome data, nine studies (90.0%) were rated as low risk and one (10.0%) as having some concerns. For measurement of the outcome, three studies (30.0%) were rated as low risk, six (60.0%) as having some concerns, and one (10.0%) as high risk. For selection of the reported result, seven studies (70.0%) were rated as low risk and three (30.0%) as having some concerns.

Overall, the methodological quality of the included studies was moderate. Most studies were judged to have some concerns regarding overall risk of bias, with the main concerns arising from deviations from intended interventions and measurement of the outcome. Some concerns were also identified in the randomization process. Detailed results are presented in Figure 2.

**Figure 2:**
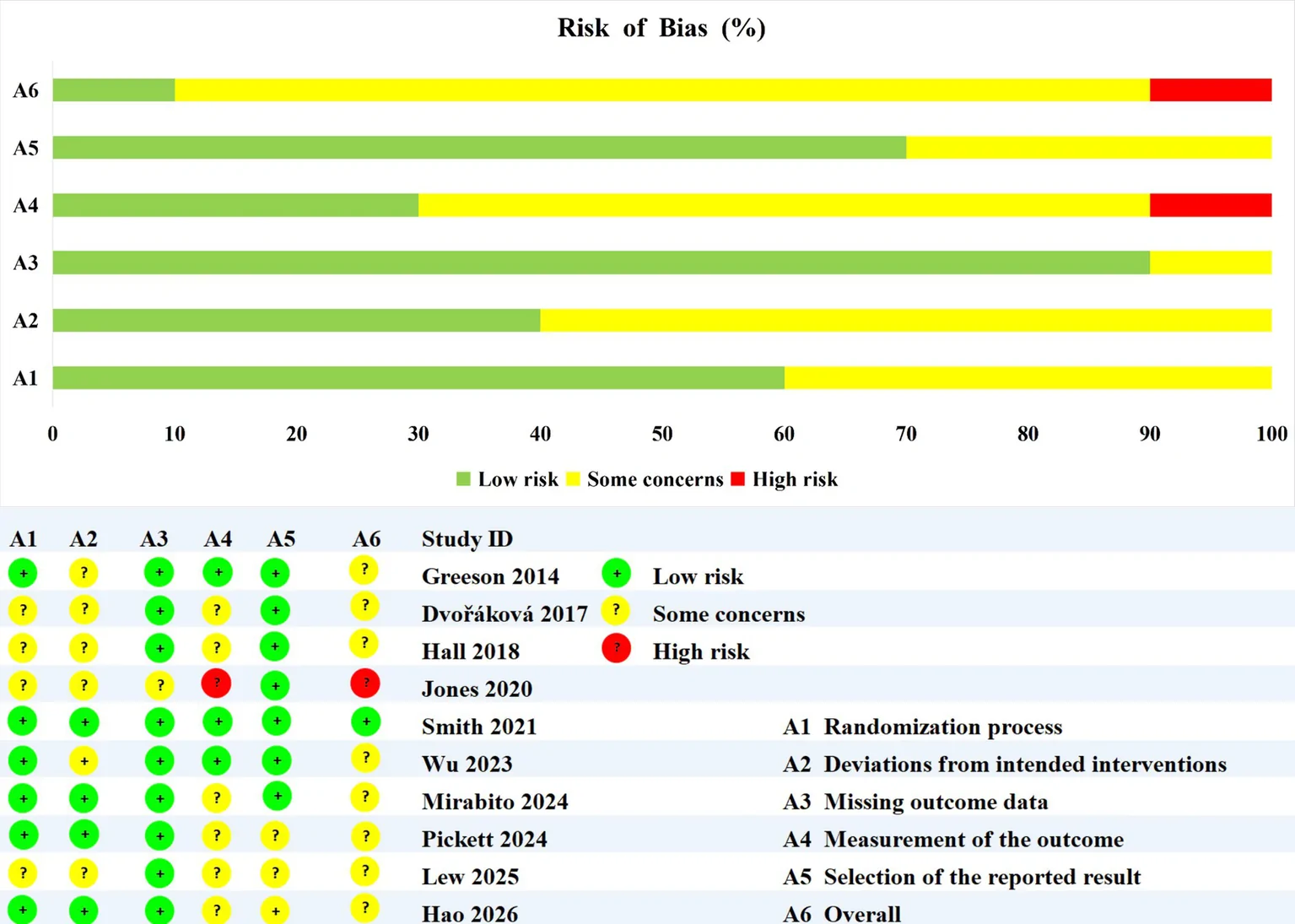
Risk-of-bias assessment of the included studies.

### 3.4 Meta-analysis results

A total of 10 RCTs were included in this meta-analysis, and a random-effects model was used. The results showed that MBIs significantly improved sleep quality in university students, with greater improvement observed in the intervention group than in the control group [SMD = −0.97, 95% CI (−1.43, −0.51), *p* < 0.001]. Substantial heterogeneity was observed among the included studies (*I*^2^ = 85.1%). The detailed results are presented in Figure 3.

**Figure 3:**
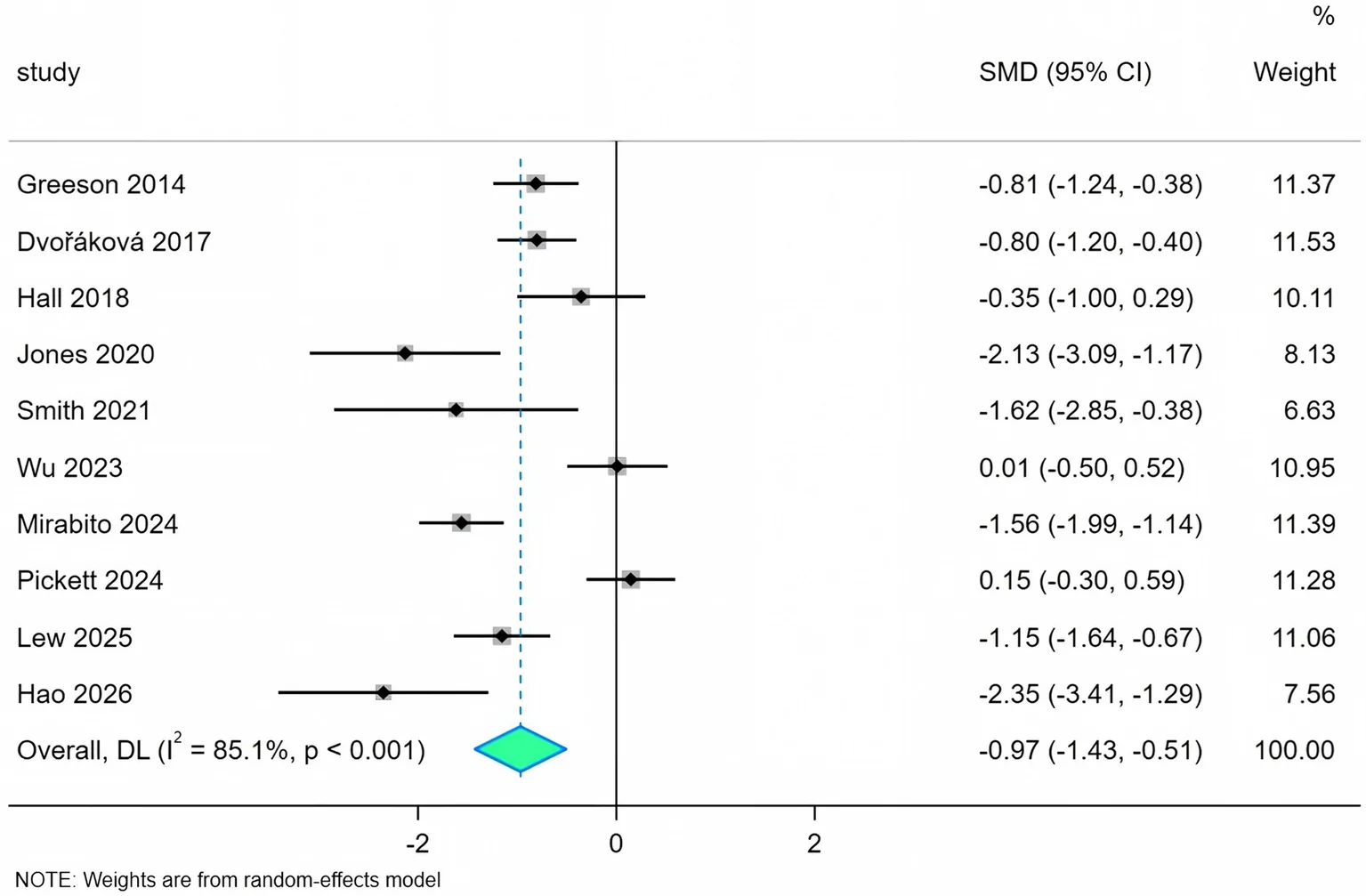
Meta-analysis of mindfulness-based interventions for improving sleep quality in university students.

### 3.5 Subgroup analysis

Given the substantial heterogeneity observed in the overall meta-analysis, subgroup analyses were conducted according to study country, mode of intervention supervision, outcome measurement instrument, intervention type, intervention duration, intervention frequency, and duration of each intervention session. These analyses were performed to further explore potential sources of heterogeneity and to compare the effects of different types and doses of MBIs on sleep quality in university students.

Subgroup analysis by study country showed that studies conducted in the United States [SMD = −1.04, 95% CI (−1.65, −0.44)] and Canada [SMD = −1.15, 95% CI (−1.64, −0.67)] reported significant improvements in sleep quality among university students, whereas the effect observed in studies conducted in China [SMD = −0.80, 95% CI (−1.93, 0.33)] was not statistically significant. No statistically significant difference in intervention effects was observed across countries (*Q* = 0.34, df = 2, *p* = 0.846), suggesting that study country was unlikely to explain the observed heterogeneity. The detailed results are presented in Figure 4.

**Figure 4:**
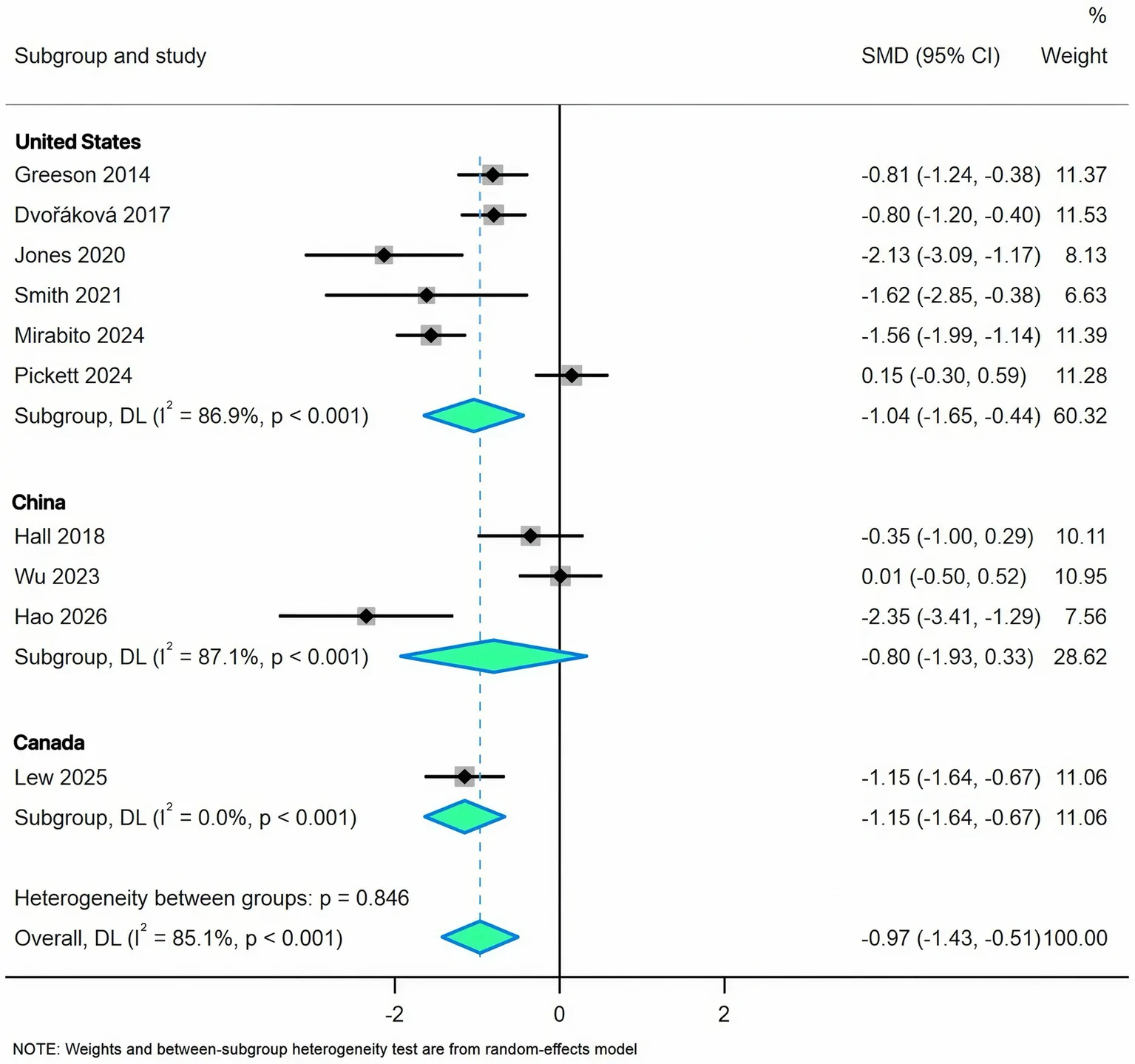
Subgroup analysis by country.

Subgroup analysis by mode of intervention supervision showed that MBIs delivered with in-person supervision [SMD = −1.08, 95% CI (−1.73, −0.42)] and online supervision [SMD = −0.86, 95% CI (−1.61, −0.12)] both significantly improved sleep quality in university students. No statistically significant difference was observed between online and in-person supervision (*Q* = 0.18, df = 1, *p* = 0.671), suggesting that supervision mode was unlikely to account for the observed heterogeneity. The detailed results are presented in Figure 5.

**Figure 5:**
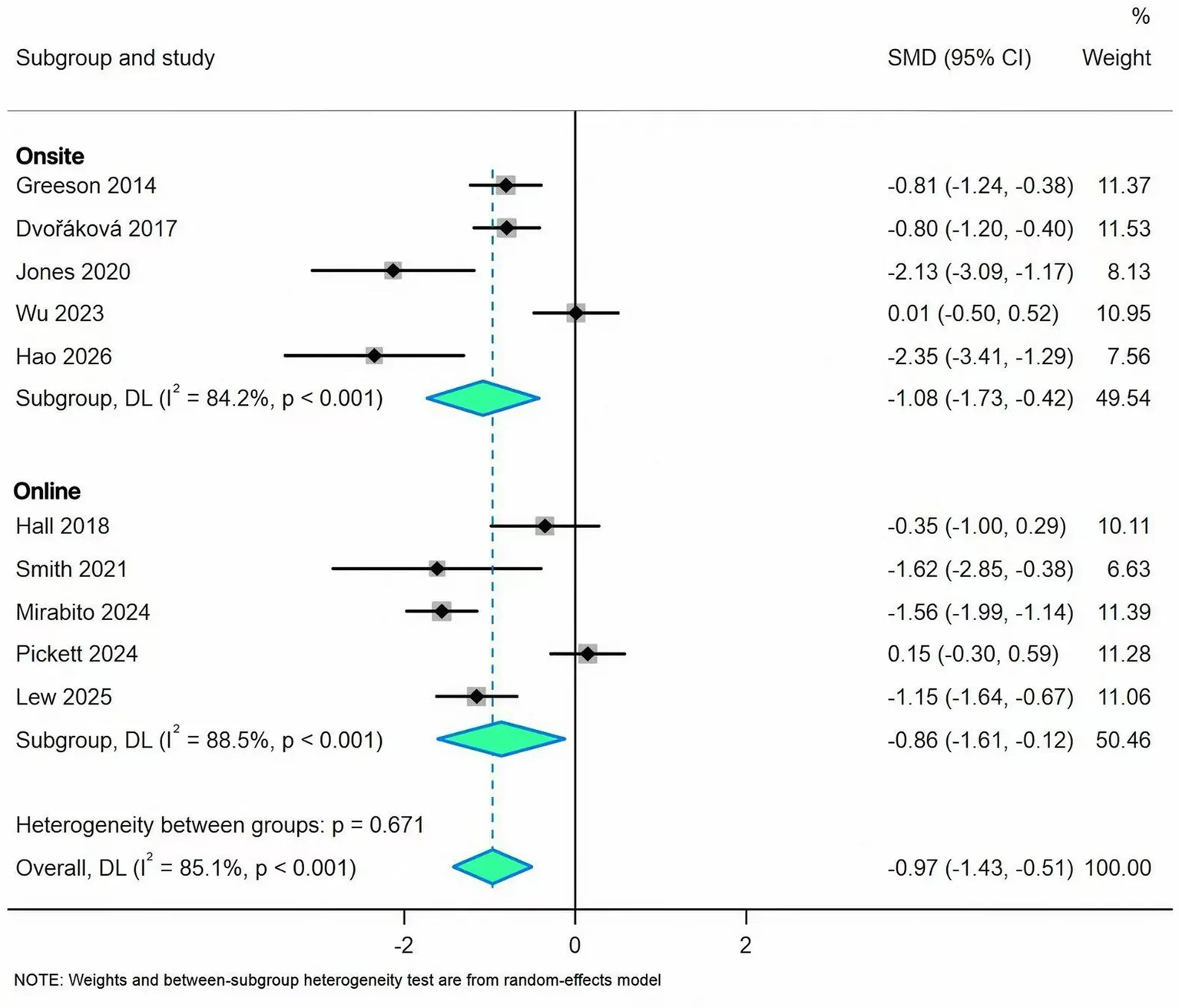
Subgroup analysis by mode of intervention supervision.

Subgroup analysis by outcome measurement instrument showed that MBIs significantly improved sleep quality in university students when assessed using the MOS SLP9 [SMD = −0.81, 95% CI (−1.24, −0.38)], PSQI [SMD = −1.11, 95% CI (−1.65, −0.56)], and PROMIS [SMD = −1.62, 95% CI (−2.85, −0.38)], whereas the improvement was not statistically significant when assessed using the SCI [SMD = 0.15, 95% CI (−0.30, 0.59)]. Further comparison of intervention effects across different outcome measurement instruments showed a statistically significant between-group difference (*Q* = 17.53, df = 3, *p* = 0.001), suggesting that the measurement instrument may partly account for the observed between-study differences in effect estimates. Because only one study was included for each of the MOS SLP9, PROMIS, and SCI subgroups, these findings should be interpreted with caution. The detailed results are presented in Figure 6.

**Figure 6:**
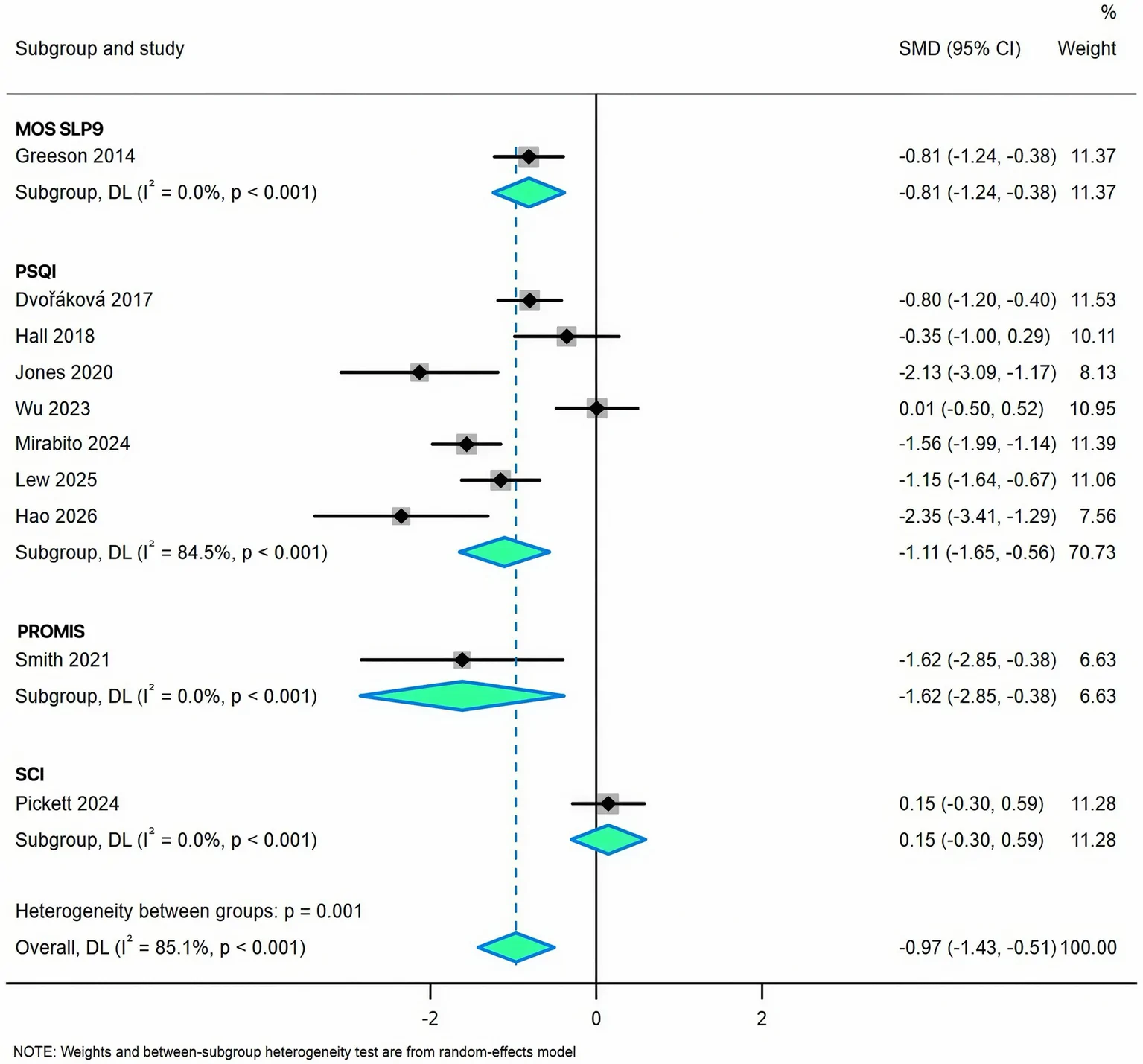
Subgroup analysis by sleep outcome measure.

Subgroup analysis by intervention type showed that MBSR [SMD = −1.22, 95% CI (−1.74, −0.71)] and MBCT [SMD = −2.35, 95% CI (−3.41, −1.29)] significantly improved sleep quality in university students, whereas the effect of mindfulness meditation [SMD = −0.50, 95% CI (−1.10, 0.11)] was not statistically significant. Further comparison of intervention effects across different intervention types revealed a statistically significant between-group difference (*Q* = 9.38, df = 2, *p* = 0.009), indicating that the effects differed significantly across types of MBIs and that intervention type may be a potential source of the overall heterogeneity. Because only one study was included in the MBCT subgroup, this finding should be interpreted with caution. The detailed results are presented in Figure 7.

**Figure 7:**
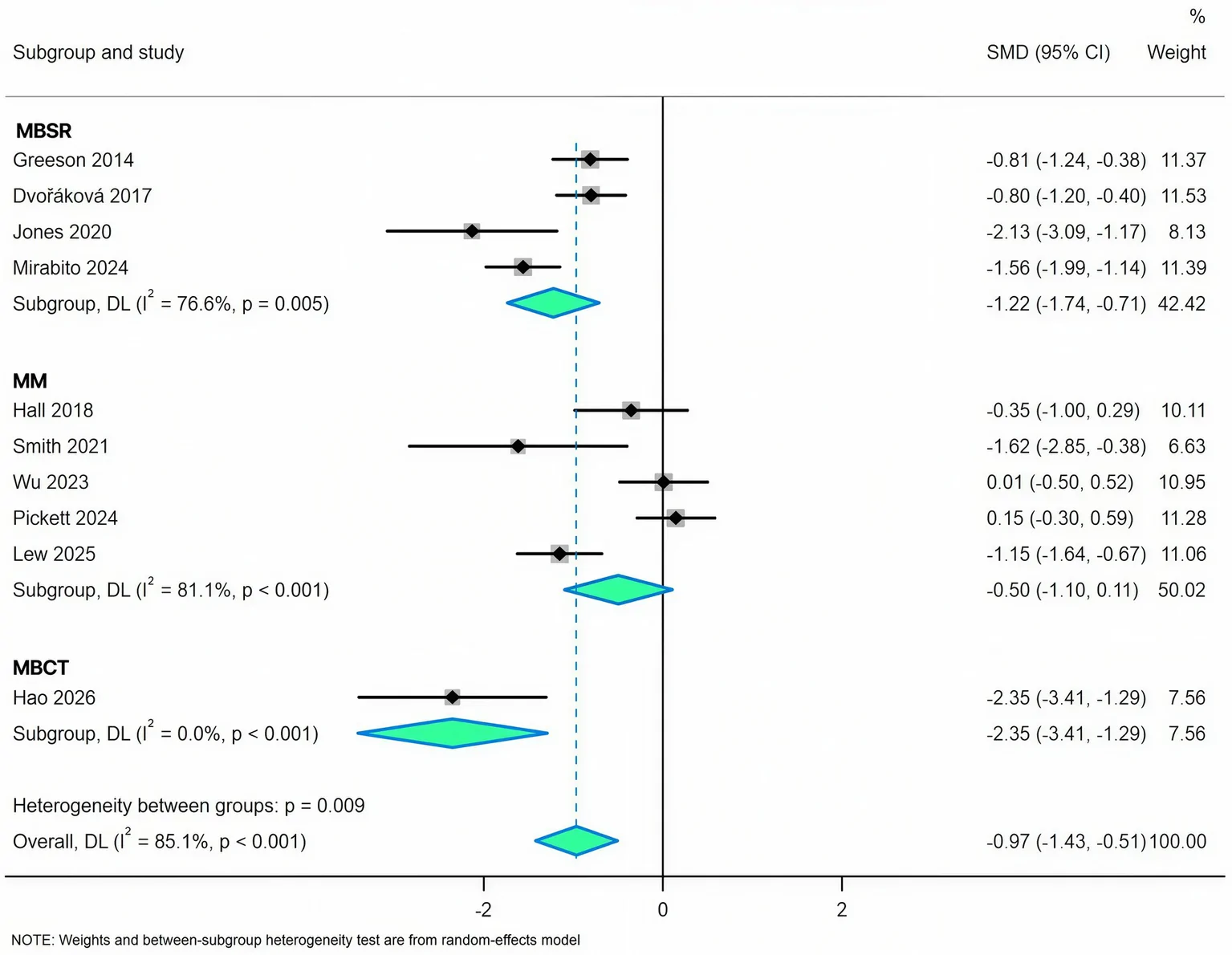
Subgroup analysis by intervention type.

Subgroup analysis was conducted according to intervention duration. Following the grouping approach used in previous relevant meta-analyses, intervention duration was categorized into two subgroups using 6 weeks as the cutoff: ≤6 weeks and >6 weeks (37). The results showed that MBIs significantly improved sleep quality in university students in both the ≤6-week subgroup [SMD = −0.70, 95% CI (−1.22, −0.18)] and the >6-week subgroup [SMD = −1.56, 95% CI (−2.63, −0.50)]. Although the magnitude of improvement was numerically greater in studies with intervention durations >6 weeks, further comparison between the two subgroups showed no statistically significant between-group difference (*Q* = 2.05, df = 1, *p* = 0.152). This indicates that intervention effects did not differ significantly by intervention duration and that intervention duration may not be a major source of the overall heterogeneity. The detailed results are presented in Figure 8.

**Figure 8:**
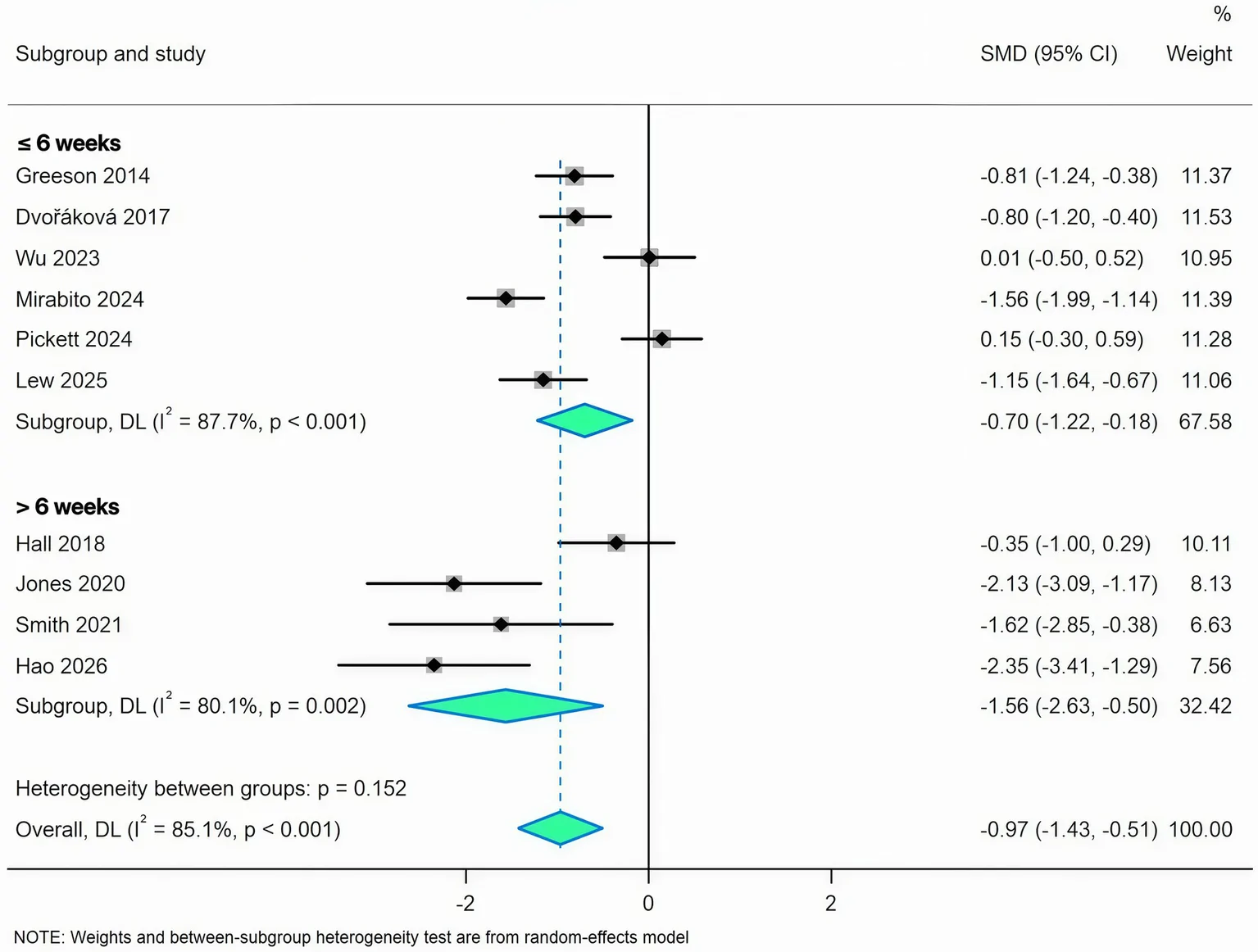
Subgroup analysis by intervention duration.

Subgroup analysis was conducted according to intervention frequency. Based on the grouping approach used in previous meta-analyses of MBIs for improving sleep quality, as well as the distribution of intervention frequencies across the studies included in the present review, intervention frequency was categorized into two subgroups: ≤1 session/week and >1 session/week (38). The results showed that MBIs significantly improved sleep quality in university students in both the ≤1 session/week subgroup [SMD = −1.22, 95% CI (−2.19, −0.26)] and the >1 session/week subgroup [SMD = −0.84, 95% CI (−1.41, −0.28)]. Further comparison of intervention effects between the two frequency subgroups showed no statistically significant between-group difference (*Q* = 0.44, df = 1, *p* = 0.506), indicating that intervention effects did not differ significantly by intervention frequency and that intervention frequency may not be a major source of the overall heterogeneity. The detailed results are presented in Figure 9.

**Figure 9:**
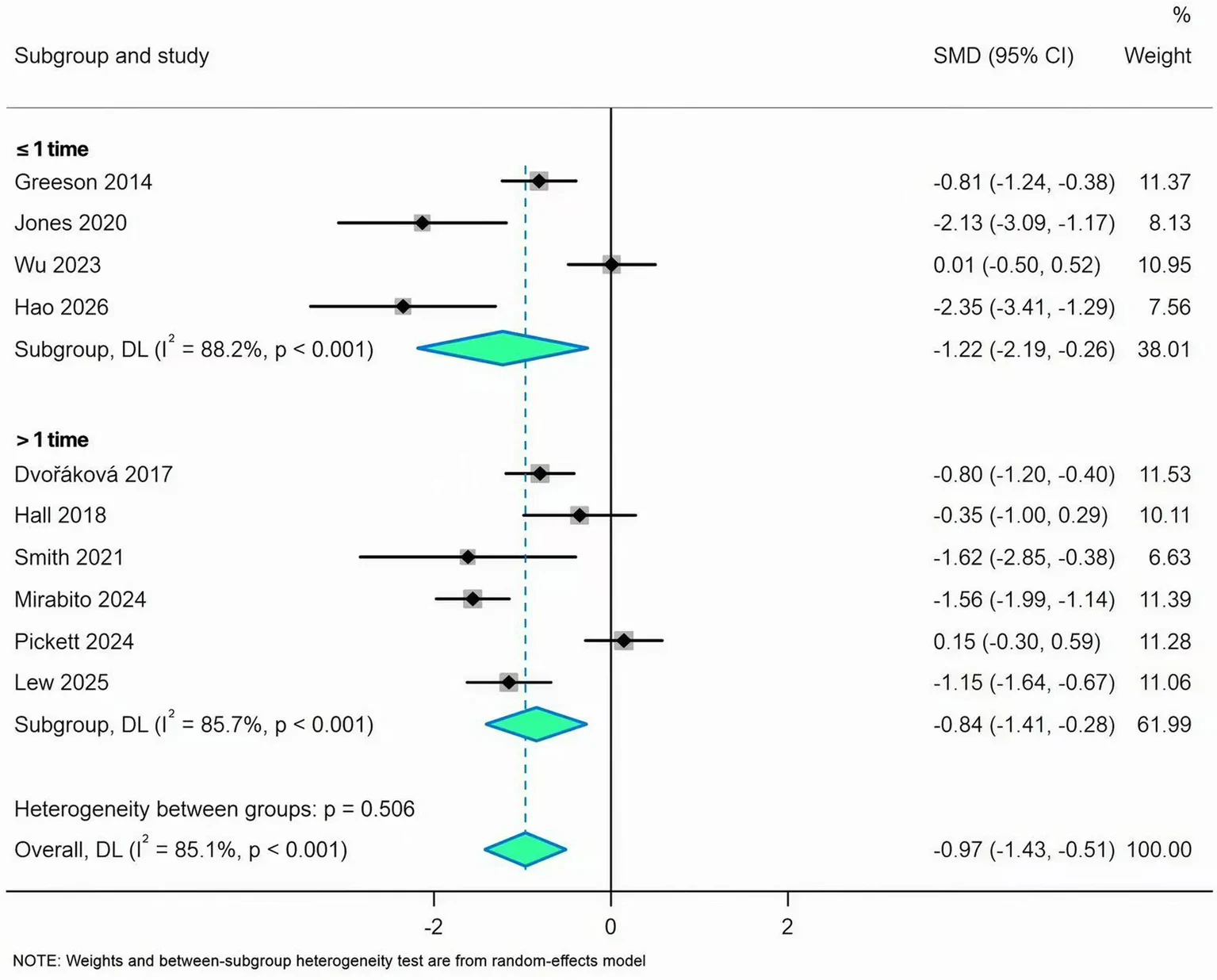
Subgroup analysis by intervention frequency.

Subgroup analysis was conducted according to the duration of each intervention session. Following the grouping approach used in previous relevant meta-analyses and considering the distribution of session durations across the studies included in the present review, session duration was categorized into two subgroups using 60 min as the cutoff: ≤60 min and >60 min (39, 40). The results showed that the improvement in sleep quality was not statistically significant in the ≤60-min subgroup [SMD = −0.50, 95% CI (−1.10, 0.11)], whereas MBIs lasting >60 min per session significantly improved sleep quality in university students [SMD = −1.39, 95% CI (−1.92, −0.86)]. Further comparison of intervention effects between the two session-duration subgroups revealed a statistically significant between-group difference (*Q* = 4.69, df = 1, *p* = 0.030), indicating that intervention effects differed significantly according to session duration and that session duration may be a potential source of the overall heterogeneity. The detailed results are presented in Figure 10.

**Figure 10:**
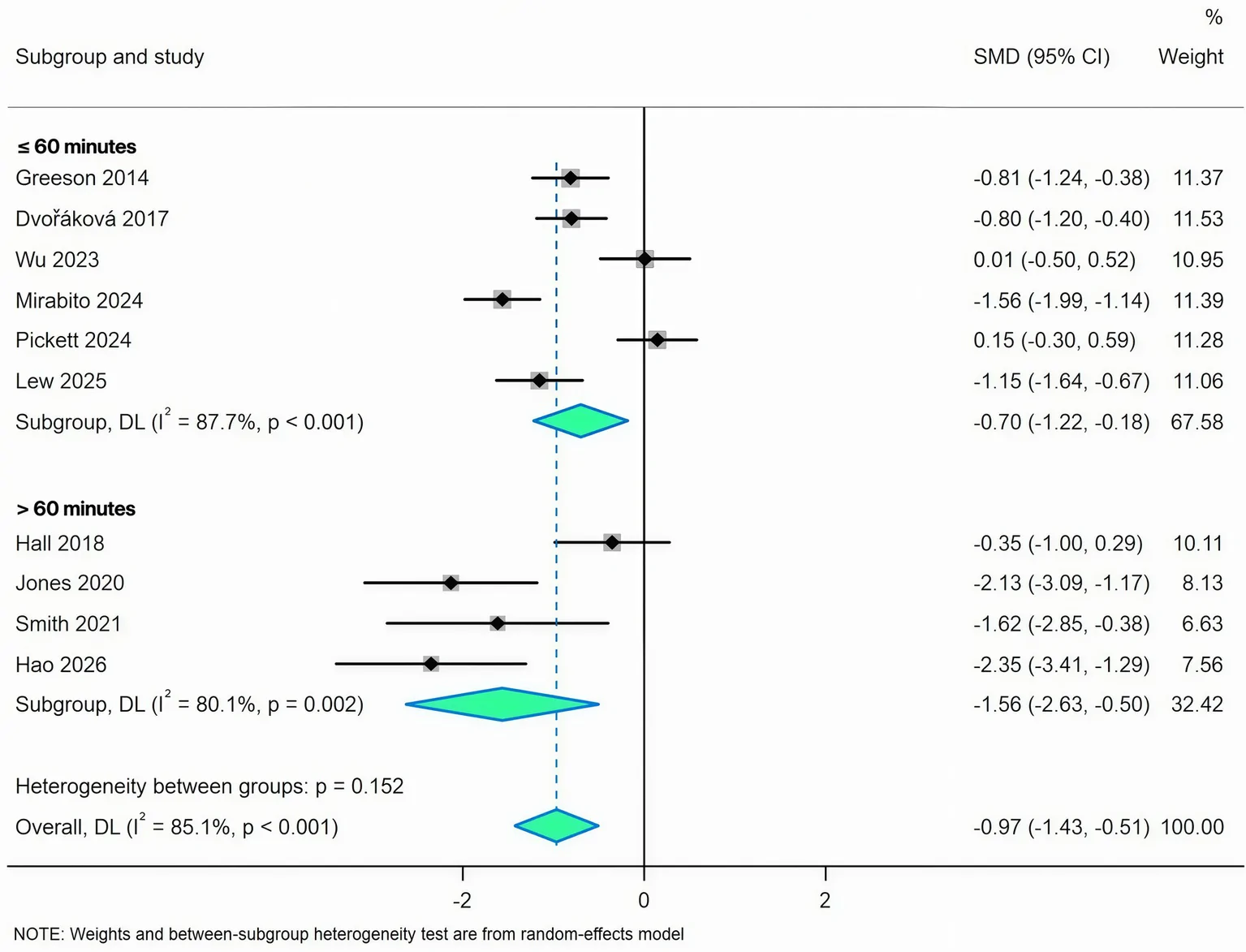
Subgroup analysis by session duration.

Taken together, the subgroup analyses showed no statistically significant between-group differences according to study country, mode of intervention supervision, intervention duration, or intervention frequency. In contrast, statistically significant between-group differences were observed for the sleep outcome measurement instrument (*p* = 0.001), intervention type (*p* = 0.009), and duration of each intervention session (*p* = 0.030), suggesting that these factors may be potential sources of between-study heterogeneity.

### 3.6 Sensitivity test and bias test

#### 3.6.1 Sensitivity test

To assess the robustness of the pooled meta-analytic results, a leave-one-out sensitivity analysis was performed by sequentially excluding each individual study. The results showed that, after the exclusion of any single study, the direction of the pooled effect estimate remained negative and the corresponding 95% CIs did not cross the line of no effect. These findings were consistent with the primary analysis, indicating that no individual study had a decisive influence on the overall effect and that the pooled results were relatively robust. The detailed results are presented in Figure 11.

**Figure 11:**
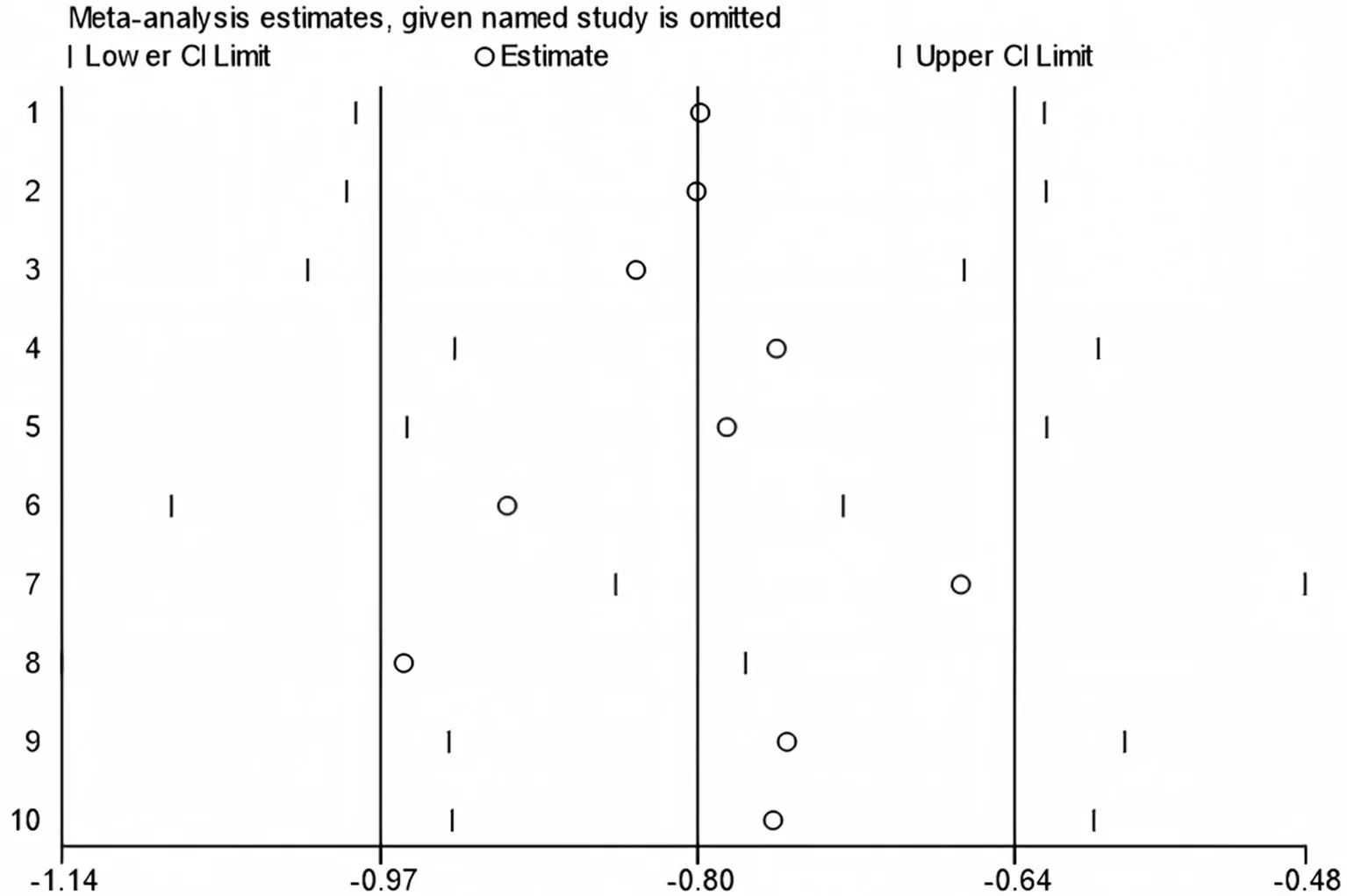
Sensitivity analysis of the effects of mindfulness-based interventions on sleep quality in university students.

#### 3.6.2 Bias risk test

Publication bias among the included studies was assessed using a funnel plot in combination with Egger’s test. The funnel plot showed some degree of asymmetry in the distribution of studies; however, further assessment using Egger’s test showed no statistically significant evidence of publication bias (*p* = 0.270). Taken together, the funnel plot and Egger’s test provided insufficient evidence of substantial publication bias among the included studies, suggesting a certain degree of reliability of the meta-analysis results. The detailed results are presented in Figure 12.

**Figure 12:**
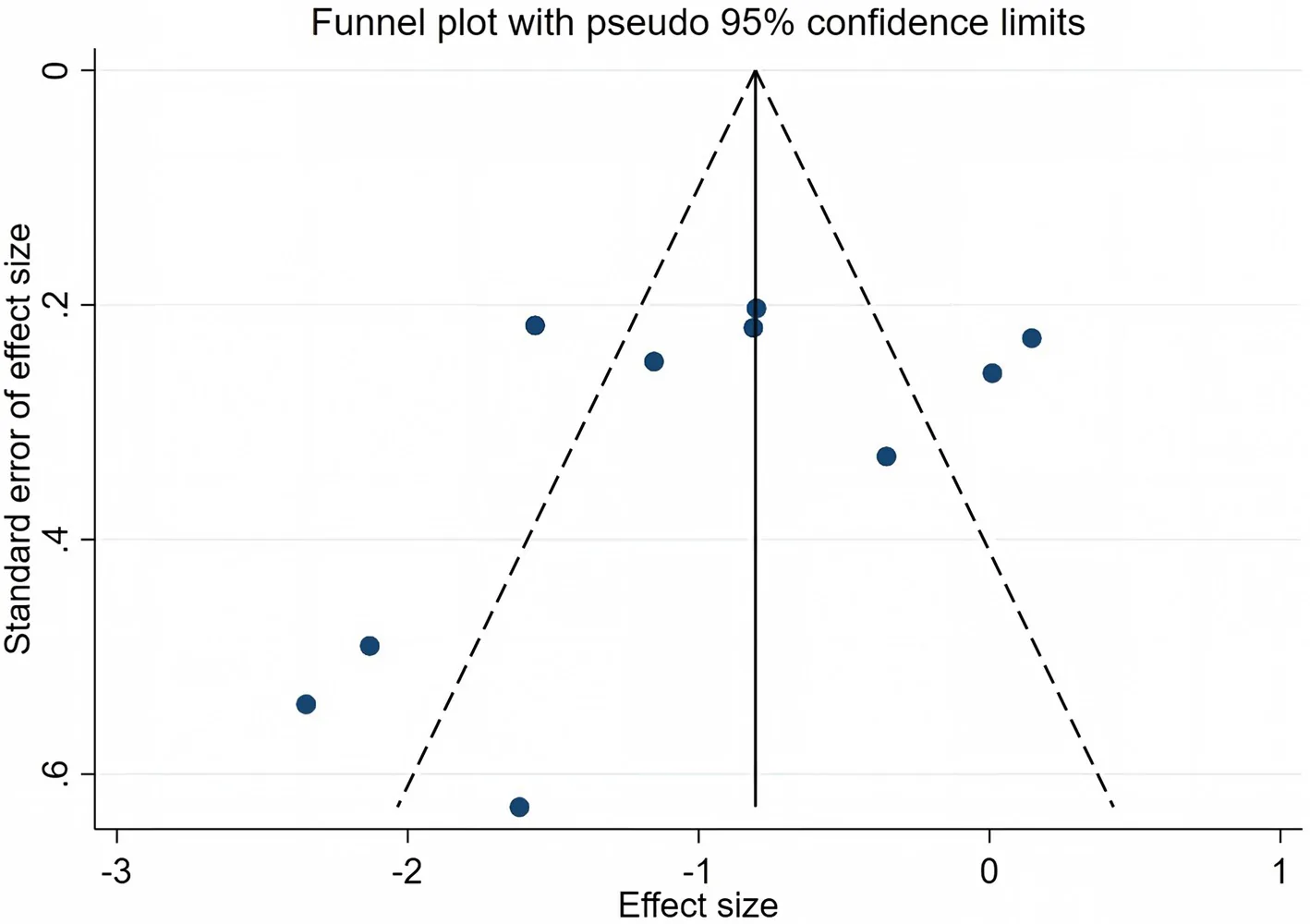
Funnel plot of publication bias for the effects of mindfulness-based interventions on sleep quality in university students.

### 3.7 Certainty of evidence assessment

The certainty of evidence regarding the effect of MBIs on sleep quality in university students was further assessed using the GRADE approach (41) (Table 3). The certainty of evidence for this outcome was rated as low. The evidence was downgraded by one level for risk of bias because most of the included studies had some concerns regarding bias and one study was rated as having a high risk of bias. It was also downgraded by one level for inconsistency because substantial between-study heterogeneity was observed (*I*^2^ = 85.1%), and the subgroup analysis showed a statistically significant difference across different outcome measurement instruments. No downgrading was applied for indirectness, imprecision, or publication bias because the populations, interventions, comparators, and sleep-related outcomes of the included studies were directly relevant to the research question, and the funnel plot combined with Egger’s test did not indicate significant publication bias (*p* = 0.270). Overall, the current evidence suggests that MBIs may improve sleep quality in university students, although the certainty of the evidence remains low.

**Table 3:** GRADE assessment of the certainty of evidence.

Certainty assessment							No. of patients		Effect	Certainty	Importance
No. of studies	Study design	Risk of bias	Inconsistency	Indirectness	Imprecision	Other considerations	Intervention	Control	Absolute (95% CI)		
10	Randomised trials	Serious	Serious	Not serious	Not serious	None	335	303	SMD 0.97 lower (1.43 lower to 0.51 lower)	⨁⨁◯◯ Low	IMPORTANT

## 4 Discussion

### 4.1 Summary of evidence

This study systematically evaluated the effects of MBIs on sleep quality in university students based on evidence from 10 RCTs involving 638 participants. The results showed that MBIs significantly improved sleep quality in university students (SMD = −0.97), providing preliminary evidence that mindfulness, as a non-pharmacological intervention, may be beneficial for promoting sleep health in promoting sleep health in university settings. However, the pooled effect should be interpreted with caution. Substantial statistical heterogeneity was observed across studies (*I*^2^ = 85.1%), indicating that the variability in effect estimates exceeded what could be explained by random error alone. This suggests that the beneficial effects of MBIs may not be entirely consistent across different study settings and may be influenced by multiple factors, including intervention protocols, intervention dose, participant characteristics, and methods of outcome assessment.

To further explore potential sources of heterogeneity, subgroup analyses were conducted according to study country, mode of intervention supervision, sleep outcome measurement instrument, intervention type, intervention duration, intervention frequency, and duration of each intervention session. Statistically significant between-subgroup differences were observed for the sleep outcome measurement instrument (*p* = 0.001), intervention type (*p* = 0.009), and duration of each intervention session (*p* = 0.030), suggesting that these factors may be potential sources of between-study heterogeneity. Notably, substantial heterogeneity remained within most subgroups, indicating that the current subgroup analyses could explain only part of the observed variation in effect estimates and that other unidentified moderating factors may also exist.

In addition, the GRADE assessment indicated that the certainty of evidence for the primary outcome was low. This was mainly attributable to concerns regarding risk of bias in most of the included studies and substantial inconsistency across studies. Nevertheless, sensitivity analyses showed that the direction of the overall effect remained unchanged after the exclusion of any single study, and Egger’s test did not indicate significant publication bias (*p* = 0.270), suggesting that the overall findings were relatively robust. Overall, the available evidence supports the possibility that MBIs may improve sleep quality in university students. However, given the substantial heterogeneity and low certainty of evidence, the magnitude of the effect, the conditions under which these interventions may be most applicable, and the optimal intervention strategy require further confirmation in high-quality studies.

### 4.2 Comparison with previous studies and potential mechanisms

This study found that MBIs significantly improved sleep quality in university students; however, this finding is inconsistent with the conclusions of most previous meta-analyses in this field. Among the relevant studies considered for comparison, only the findings of Zuo et al. were consistent with those of the present study. In their evaluation of the effects of MBIs on mental health among university students, Zuo et al. found that such interventions significantly improved sleep quality (16). In contrast, Dawson et al., in a systematic review of the effects of MBIs on the physical and mental health of university students, reported that although these interventions improved several mental health outcomes, including stress, anxiety, and depression, the improvement in sleep outcomes did not reach statistical significance (42). Subsequently, Pan et al. focused specifically on the effects of MBSR on mental health and psychological quality of life among university students and likewise found no significant improvement in sleep quality (15). More recently, Tadros et al. conducted a systematic review of psychological interventions for improving sleep quality in university students, and the pooled effect for the mindfulness-based intervention subgroup was also not statistically significant (14).

The discrepancies among these findings may be explained primarily by two factors. First, previous meta-analyses included relatively few studies in pooled analyses of sleep outcomes, generally ranging from two to five studies. Even the meta-analysis by Tadros et al., which included the largest number of relevant studies, incorporated only five studies examining MBIs and sleep outcomes (14), In contrast, the present study included 10 RCTs, thereby expanding the direct evidence base regarding the effects of MBIs on sleep quality in university students. Second, most previous meta-analyses were not specifically designed to investigate sleep quality but instead evaluated the effects of MBIs across multiple domains of physical and mental health in university students. For example, Dawson et al. assessed a range of outcomes, including stress, anxiety, depression, wellbeing, rumination, and sleep, with sleep representing only one of several outcomes examined (42). By contrast, the present study focused specifically on sleep quality in university students and systematically synthesized a larger body of randomized controlled trial evidence, thereby providing a more focused evaluation of the potential sleep-related benefits of MBIs.

To further understand the potential pathways through which MBIs may improve sleep quality in university students, potential mechanisms can be considered from both physiological and psychological perspectives. It should be noted that the present study primarily evaluated the overall effects of MBIs on sleep quality and did not systematically assess relevant physiological or psychological mechanisms. Therefore, the following mechanistic interpretations are based mainly on existing theoretical frameworks and empirical evidence.

From a physiological perspective, some university students may exhibit higher cortisol levels during periods of academic or examination stress (43). Studies have also found that higher cortisol levels are associated with poorer sleep quality among student populations (44), suggesting that stress-related neuroendocrine activity may be associated with sleep disturbances. Notably, a meta-analysis showed that stress-management interventions, including mindfulness, may influence cortisol levels (45). Based on the above evidence, it is reasonable to speculate that mindfulness-based interventions may improve sleep quality, at least to some extent, by modulating stress-related endocrine responses. From a psychological perspective, MBIs may indirectly improve sleep by alleviating symptoms of anxiety and depression in university students. Previous studies have shown that anxiety and depression are closely associated with sleep quality in this population, with more severe symptoms generally corresponding to poorer sleep quality (46). MBIs have been shown to significantly reduce depressive symptoms while also improving insomnia symptoms among university students (47). Other research has similarly found that reductions in anxiety and stress following MBIs may be accompanied by improvements in sleep quality (36). Therefore, alleviating negative emotional states such as anxiety and depression may represent one important psychological pathway through which MBIs improve sleep quality in university students.

Overall, compared with previous research, the present study included a larger number of RCTs reporting focused specifically on sleep quality, thereby providing additional evidence supporting the potential effectiveness of MBIs for improving sleep quality in university students and helping to clarify inconsistencies in the existing literature. The potential mechanisms underlying these effects may involve reductions in physiological stress and pre-sleep arousal, as well as alleviation of negative emotional states such as anxiety and depression. However, these mechanisms remain largely theoretical and inferential on the basis of existing evidence and should be further examined through studies incorporating physiological measurements and mediation analyses.

### 4.3 Clinical and practical implications

The findings suggest that MBIs may represent a potential non-pharmacological approach to improving sleep health among university students. Compared with pharmacological treatments, mindfulness training offers greater flexibility in delivery and can be integrated with mental health education and stress management programs, making it particularly suitable for implementation in university settings. For students experiencing sleep difficulties who prefer non-pharmacological approaches to improve sleep, mindfulness training may be considered as an adjunctive strategy for sleep health promotion.

The subgroup analyses also provide some guidance for optimizing mindfulness-based intervention programs and their practical implementation in university settings. This study explored several factors that may influence improvements in sleep quality, including mode of supervision, intervention duration, intervention frequency, intervention type, and duration of each intervention session. No significant between-group differences were observed according to supervision mode, intervention duration, or intervention frequency, suggesting some flexibility in the delivery and scheduling of MBIs. In contrast, potential differences were observed across intervention types and session durations, indicating that these factors may warrant greater attention when optimizing future intervention programs.

With regard to intervention type, this study found statistically significant differences in sleep-related effects across different types of MBIs, suggesting that intervention type may influence the sleep benefits experienced by university students. Previous studies have also reported differences in effect estimates among various forms of MBIs. Chan et al. found that MBCT, MBSR, and MM were associated with different levels of improvement in sleep outcomes, showing a pattern broadly similar to that observed in the present study. However, that study primarily compared each intervention type with a control condition and did not further test between-group differences across intervention types (48). In contrast, studies by Rusch et al. and Choukas et al. indicated that although MBIs may improve sleep, there is currently insufficient evidence to demonstrate that any single intervention modality has a consistent advantage over others (49, 50). Therefore, the differences in intervention effects observed in the present study should be interpreted with caution. In particular, the MBCT subgroup included only one study, and its relatively large effect estimate may have been influenced by the characteristics of that individual study and its specific intervention protocol. Based on the current evidence, it remains unclear whether MBCT, MBSR, or MM offers a clear advantage over the others. Universities should therefore consider student needs, intervention goals, and implementation conditions when selecting a specific mindfulness-based intervention.

With regard to session duration, the present study further found that the >60 min subgroup showed a more pronounced improvement in sleep quality, suggesting that the duration of each intervention session may be a potential factor influencing the effectiveness of MBIs. Previous studies have also provided evidence linking the amount of time devoted to mindfulness practice with sleep-related benefits. A meta-analysis by Lee and Park found a positive association between daily mindfulness practice time and improvements in sleep outcomes (51), while a study by Liu et al. among undergraduate students also reported an association between daily mindfulness practice time and improvements in PSQI scores (52). These findings suggest that greater engagement in mindfulness practice may contribute to greater sleep-related benefits. However, the finding for sessions lasting >60 min was derived from between-study subgroup comparisons, and session duration may also be associated with intervention type, total training dose, and level of instructional intensity. Therefore, this finding should be interpreted as preliminary guidance for the design of mindfulness-based intervention programs rather than as evidence that sessions lasting >60 min are optimal.

Overall, when implementing MBIs for sleep health in university settings, the mode of delivery may be selected flexibly according to intervention goals, available course resources, and student characteristics, while taking feasibility and student acceptability into account. Particular attention may be given to the appropriateness of intervention type and session duration to enhance the applicability and practical value of intervention programs.

### 4.4 Limitations and future research directions

Although this study systematically evaluated the effects of MBIs on sleep quality in university students and yielded several relevant findings, several limitations should be acknowledged. First, the number of included studies and the overall sample size were relatively limited, and substantial between-study heterogeneity was observed. Only 10 RCTs were included, several of which had small sample sizes, and the overall heterogeneity was high (*I*^2^ = 85.1%), which may affect the stability of the pooled effect estimate. Second, the geographical representation of the included studies was limited. Most studies were conducted in the United States, China, and Canada, with limited evidence available from university students in other countries and cultural settings. Therefore, the generalizability of the findings across different regions and cultural contexts requires further investigation. Third, only studies published in English were included. Relevant studies published in other languages may therefore have been missed, potentially introducing language bias and limiting the comprehensiveness of the evidence base. Finally, sleep outcomes were assessed primarily using subjective measures. The included studies mainly used self-reported instruments such as the PSQI, MOS SLP9, PROMIS, and SCI, with limited use of objective sleep measures such as actigraphy and polysomnography. As a result, the findings may have been influenced by subjective perceptions and reporting bias, and the effects of MBIs on objectively measured sleep quality remain to be clarified.

Future research should address these limitations. First, high-quality RCTs with larger sample sizes and multicenter designs are needed. Greater standardization of intervention type, intervention dose, and outcome assessment methods should also be considered to improve the precision of effect estimates and facilitate the identification of sources of between-study heterogeneity. Second, future studies should include university students from a broader range of geographical and cultural backgrounds, including different countries, regions, and educational settings, to examine the applicability and generalizability of MBIs across diverse cultural contexts. Finally, objective sleep measures, such as actigraphy and polysomnography, should be incorporated alongside subjective sleep questionnaires. Assessing multiple dimensions of sleep, including sleep duration, sleep efficiency, and nocturnal awakenings, through a combination of subjective and objective measures may provide a more comprehensive evaluation of the effects of MBIs on sleep quality in university students.

## 5 Conclusion

MBIs may represent a promising non-pharmacological approach to improving sleep quality among university students and may serve as an adjunctive strategy for sleep health promotion in university settings. Nevertheless, the current body of evidence is constrained by the limited number of eligible studies and modest aggregate sample sizes. Further high-quality, large-scale, multicenter RCTs incorporating objective sleep measures are needed to confirm the effects of MBIs on sleep quality.

## Data Availability

The original contributions presented in the study are included in the article/supplementary material, further inquiries can be directed to the corresponding author.

## Footnotes

1
https://www.crd.york.ac.uk/PROSPERO/view/CRD420261458068
